# Awareness, Treatment, and Control of Hypertension Among Hypertensive Older Adults in Iran: A Cross‐Sectional Study From the Ardakan Cohort Study on Aging

**DOI:** 10.1002/hsr2.71660

**Published:** 2025-12-15

**Authors:** Sina Kazemian, Fatemeh‐sadat Tabatabaei, Amirali Azimi, Isa Akbarzade, Elham Hooshmand

**Affiliations:** ^1^ Cardiac Primary Prevention Research Center, Cardiovascular Diseases Research Institute Tehran University of Medical Sciences Tehran Iran; ^2^ Tehran Heart Center, Cardiovascular Diseases Research Institute Tehran University of Medical Sciences Tehran Iran; ^3^ Iranian Research Center on Aging University of Social Welfare and Rehabilitation Sciences Tehran Iran

**Keywords:** aged, awareness, control, hypertension, treatment

## Abstract

**Background and Aims:**

Hypertension is a major global health concern, particularly among older adults; however, comprehensive data on hypertension management in this population remain limited in Iran. This study aims to investigate factors associated with hypertension awareness, treatment, and control in adults aged 50 and older in Ardakan City, Iran.

**Methods:**

This cross‐sectional study utilized data from the 2020 Ardakan Cohort Study on Aging, a subset of the Iranian Longitudinal Study on Aging. A stratified random sampling method was used to recruit 2333 hypertensive participants aged 50 years or older from the general population. Data were collected through validated questionnaires, medical interviews, and standardized blood pressure measurements. Prevalence estimates of hypertension awareness, treatment, control through lifestyle modifications, and control among treated individuals were calculated. Multivariable logistic regression models were applied to identify independent predictive factors while adjusting for potential confounding variables.

**Results:**

Participants (mean age: 62.8 ± 7.6 years; 48.7% women) demonstrated varying prevalence estimates for hypertension management: 84% were aware, 81% were treated with anti‐hypertensive medication, and 60% achieved blood pressure control. Women exhibited a significantly higher prevalence of hypertension management than men, including awareness (52.8% vs. 47.2%, *p* < 0.001), treatment (52.2% vs. 47.8%, *p* < 0.001), control through lifestyle modifications (57.0% vs. 43.0%, *p* < 0.001), and control among treated (56.5% vs. 42.1%, *p* < 0.001). Multivariable analysis identified poor physical condition and depressive symptoms as independent predictors of higher hypertension awareness.

**Conclusion:**

This study highlights the need for targeted interventions to improve hypertension management among adults aged 50 years or older, as only slightly over half of treated individuals achieve the blood pressure control goal. Addressing both physical and mental health aspects is critical for enhancing hypertension outcomes in this population.

## 1 Introduction

Hypertension is a global health concern that increases the risk of cardiovascular diseases, stroke, and other chronic diseases [1]. Its prevalence is projected to reach 29.2% worldwide by 2025—over 2 billion people—with more than two‐thirds of the population living in low‐ and middle‐income countries (LMICs) [2]. The risk of hypertension increases with age, making it a critical issue for older adults. In Iran, approximately 30% of adults aged 50 years and older, and around 50% of those aged 70 and older, are estimated to have hypertension [3, 4]. Raising awareness, ensuring optimal treatment, and improving control of this condition are crucial for more effective disease management and reducing healthcare costs [5].

Comprehensive data on the awareness, treatment, and control of hypertension among older adults in Iran are lacking despite its high prevalence. Previous studies have reported varying estimates of hypertension awareness, ranging from 49.7% to 68.2%, and treatment prevalence ranging from 53.5% to 80.0% in Iran, with higher prevalence observed among older adults [3, 6, 7]. Various factors such as age, sex, socioeconomic status, education, and access to healthcare are recognized as key determinants of hypertension management [6, 8]. However, the relationship between these factors and their influence on hypertension awareness and control warrants further investigation.

The importance of managing hypertension through awareness, treatment, and control is evident, but achieving optimal treatment is challenging due to its often asymptomatic nature. This issue is particularly bolded in LMICs, where access to routine cardiovascular surveillance and affordable healthcare services is limited. Older adults face additional challenges, including polypharmacy, ineffective doctor‐patient communication, and cognitive decline, which hinder optimal treatment and increase mortality rates and the overall healthcare burden [4, 6, 8, 9, 10]. These disparities underscore the need for targeted interventions to improve hypertension management in this population.

This study aims to investigate factors associated with hypertension awareness, treatment, and control among adults aged 50 years and older in Ardakan City, Iran. Additionally, it evaluates age‐ and sex‐based disparities across six age groups and between men and women. Multivariable logistic regression models were applied to identify predictive factors, such as physical activity and depressive symptoms, while adjusting for potential confounding variables.

## 2 Methods

### 2.1 Study Design and Participants

This cross‐sectional study utilized data from the recruitment phase of the Ardakan Cohort Study on Aging (ACSA). Our study adheres to the strengthening the reporting of observational studies in epidemiology (STROBE) guidelines [11]. The Research and Ethics Committee of the University of Social Welfare and Rehabilitation Sciences approved the study protocol (IR.USWR.REC.1394.490). Every participant provided written informed consent before enrollment.

The ACSA is a subset of a Persian cohort named the Iranian Longitudinal Study on Aging (IRLSA). The IRLSA provides an in‐depth analysis of aging and related factors in Iran. Details of its study protocol have been previously published [12]. In summary, the ACSA is a population‐based cohort study of adults aged 50 years or older, which started in 2020 in Ardakan City, located in the northern part of Yazd province, Iran. The study participants were selected using a stratified random sampling method from 5196 households. To ensure a representative sample, a two‐stage stratified random sampling method was employed. First, the population was stratified based on health center regions, ensuring that each center's proportional contribution was determined. Subsequently, within each stratum, simple random sampling was conducted to select individuals. If a selected individual was married and their spouse was also aged 50 years or older, the spouse was also invited to participate in the study. This cohort's objective was to investigate various health‐related aspects of older adults, with a particular emphasis on the social determinants of aging. Information was gathered through in‐person interviews and household visits. Eligibility criteria included being at least 50 years, holding Iranian citizenship, and having resided in Ardakan City for at least 1 year. Individuals with dementia (assessed using the abbreviated mental test scores), cognitive impairments (determined by the mini‐mental state examination questionnaire), or those requiring specialized care within the last 12 months were deemed ineligible and excluded from the study.

### 2.2 Data Collection and Questionnaires

We interviewed participants using standardized and validated questionnaires designed to collect data on demographic characteristics, socioeconomic conditions, lifestyle habits, metabolic indicators, behavioral factors, comorbidities, and medical histories. Participants were asked about their history of hypertension and their use of anti‐hypertensive medications separately. Blood pressure (BP) measurements were conducted in a quiet and controlled environment to ensure accuracy and reliability. Participants were instructed to rest for 5 min prior to the measurement. During the procedure, participants were seated comfortably with proper back support, and their left arm was positioned at heart level. BP measurements were taken using the Exacta aneroid twin‐tube sphygmomanometer (Rudolf Riester GmbH, Jungingen, Germany) with an appropriately sized brachial cuff. Two measurements were recorded, 5 min apart, and the second measurement was used for statistical analysis.

We utilized several questionnaires: (i) An economic status checklist was used to gather information on participants' financial standing. They were instructed to evaluate their own status and choose from five options: high, medium to high, medium, low to medium, or low. (ii) The Health‐Related Quality Of Life Short Form (HRQoL SF‐12) was employed to investigate the quality of life related to health [13]. This tool, which has been previously translated and validated into Persian, utilizes weighted subscales to evaluate the Physical Component Summary (PCS) and Mental Component Summary (MCS) [14]. The MCS encompasses aspects such as vitality, social interaction, emotional roles, and mental well‐being, whereas the PCS covers general health, physical functioning, physical roles, and physical discomfort. Each subscale is scored from 0 to 100, with a higher score indicating better HRQoL. (iii) The Satisfaction With Life Scale (SWLS) was used to assess overall cognitive evaluations of life satisfaction. The SWLS consists of five questions, and respondents express their level of agreement or disagreement for each statement on a 7‐point scale, where 7 signifies “strongly agree” and 1 denotes “strongly disagree” [15]. The SWLS is a reliable tool, demonstrating both consistency over time and sensitivity to changes in life satisfaction. We utilized a Persian‐translated and validated version of the SWLS to assess individuals' overall life satisfaction based on their own perceptions [16]. (iv) The Hospital Anxiety and Depression Scale (HADS) was designed to evaluate psychological distress in hospital settings. The HADS consists of 14 questions, with seven assessing anxiety and seven assessing depression. Respondents rated each statement on a 4‐point scale ranging from 0 (absent) to 3 (significant) [17]. The Persian‐translated and validated version of the HADS has demonstrated strong reliability and accuracy in evaluating individuals' levels of anxiety and depression [18]. (v) The Center for Epidemiologic Studies Depression Scale (CES‐D10) was employed for depression screening [19]. This tool employs a 4‐point Likert scale, with response options spanning from “rarely or never” to “most of the time or always”, based on the frequency of depressive symptoms over the past week. We utilized the Persian‐translated and validated version of this tool [20]. The scoring system provides insight into participants' depressive states, with higher scores signifying more frequent depressive symptoms. The validity and reliability of this tool have been confirmed in prior studies [20].

### 2.3 Definitions

All BP readings were divided into five categories: (1) Normal BP (systolic blood pressure (SBP) < 120 mmHg and diastolic blood pressure (DBP) < 80 mmHg), (2) elevated BP (SBP 120–129 mmHg and DBP < 80 mmHg), (3) isolated systolic hypertension (SBP ≥ 130 mmHg and DBP < 80 mmHg), (4) isolated diastolic hypertension (SBP < 130 mmHg and DBP ≥ 80 mmHg), (5) systolic‐diastolic hypertension (SBP ≥ 130 mmHg and DBP ≥ 80 mmHg) [21]. Hypertension was defined as blood pressure measurements with SBP ≥ 130 mmHg and/or DBP ≥ 80 mmHg, a previous diagnosis of hypertension, or current use of anti‐hypertensive medications. To evaluate hypertension awareness and treatment, we assessed each participant's self‐reported diagnosis and use of anti‐hypertensive medication. Furthermore, controlled blood pressure was defined as SBP < 130 mmHg and DBP < 80 mmHg achieved either through lifestyle modifications or anti‐hypertensive medication. A previous history of diabetes mellitus was defined as a self‐reported diagnosis of diabetes or the use of glucose‐lowering drugs, while dyslipidemia was identified by a prior diagnosis or the use of lipid‐lowering medication. Individuals who had smoked at least 100 cigarettes in their lifetime but had ceased by the time of the interview were classified as former smokers [22].

Using the HRQoL SF‐12 questionnaire, we defined poor physical condition as PCS‐12 ≤ 50, and clinical depression as MCS‐12 ≤ 42 [13]. SWLS scores were categorized into seven groups: extremely satisfied (score: 31–35), satisfied (score: 26–30), slightly satisfied (score: 21–25), neutral (score: 20), slightly dissatisfied (score: 15–19), dissatisfied (score: 10–14), and extremely dissatisfied (score: 5–9) [15]. Furthermore, HADS scores were classified into three categories: normal (HADS ≤ 7), borderline‐mild (HADS 8–10), and abnormal (HADS ≥ 11) [18]. Lastly, depressive symptoms, as assessed by the CES‐D, were defined as CES‐D ≥ 10 [20].

### 2.4 Statistical Analysis

Categorical variables were reported as numbers and proportions (%), and *χ* ^2^ test was used for comparative analysis. Continuous variables with a normal distribution were reported as mean ± standard deviation and compared with the independent samples *t*‐test. All scale variables were tested using the Kolmogorov–Smirnov test and checked visually using histograms. We used binary logistic regression analysis to test the association between each questionnaire and outcomes, such as awareness, treatment, control, and control among treated individuals, adjusting for potential confounders. Variables with *p* < 0.05 in univariate analysis or those of clinical significance were recognized as potential confounders and included in the multivariable model (Awareness model adjusted for: age, sex, BMI, education level, DM, dyslipidemia; treatment model: age, sex, BMI, DM; control model: age, sex, employment status; control among treated model: age, sex, employment status). Eventually, we reported the odds ratio (OR), 95% confidence interval (95% CI), and *p* value for each questionnaire included in the models. All analyses were conducted using STATA version 14.1 (StataCorp, College Station, TX, USA), with a significance threshold set at a two‐sided *p* value of < 0.05.

## 3 Results

The initial cohort recruitment phase yielded a total of 4398 participants from the general population aged ≥ 50 years. However, after applying the eligibility criteria, 278 individuals were excluded due to conditions such as dementia (148 participants), cognitive impairment (125 participants), and advanced care needs (five participants). Additionally, for the purpose of this study, we focused on individuals with hypertension, resulting in a final sample size of 2333 participants. The mean age of the included participants was 62.8 ± 7.6 years, and 48.7% (1136 participants) were women. The participants' mean body mass index (BMI) was 29.4 ± 4.9 kg/m^2^. The most prevalent comorbidities were dyslipidemia (*N* = 1445, 61.9%), diabetes mellitus (*N* = 923, 39.8%), and coronary heart disease (*N* = 130, 5.6%), respectively. Blood pressure readings showed that the most common form of hypertension was systolic‐diastolic hypertension (47%), followed by isolated diastolic hypertension (32%), and isolated systolic hypertension (21%).

### 3.1 Awareness, Treatment, Control

**Figure 1:**
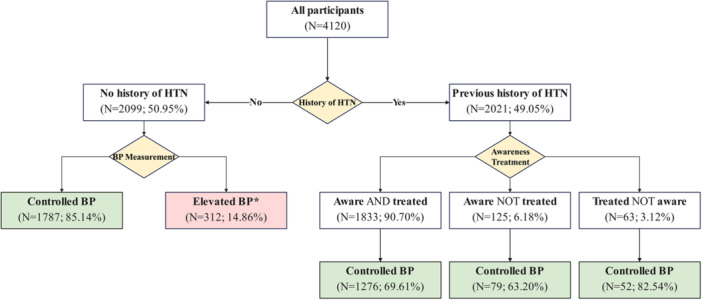
Flow chart representing an overview of blood pressure measurements and hypertension awareness, treatment, and control in the whole cohort population.

In our study, we found that approximately 84% of individuals diagnosed with hypertension were aware, 81% were receiving treatment, and 60% had achieved target blood pressure levels through lifestyle modifications or anti‐hypertensive medication. In more detail, the majority of participants with a history of hypertension demonstrated awareness and were receiving treatment (*N *= 1833; 90.70%), while 69.61% had attained the blood pressure control goal. Additionally, 312 individuals (14.86%) without a prior diagnosis of hypertension exhibited elevated blood pressure readings (Figure 1).

In terms of baseline characteristics, higher awareness, treatment, and control through lifestyle modifications prevalences were significantly associated with women, single individuals, those living alone, retired or unemployed individuals, those receiving unofficial care, and those with a history of diabetes mellitus or dyslipidemia. Furthermore, individuals who had never smoked exhibited significantly higher prevalence estimates in all three categories. Moreover, individuals with higher BMI, lower education levels, and a history of chronic kidney disease had higher awareness and treatment prevalence. Other comorbidities, such as diabetes and coronary heart disease, were only linked to higher treatment prevalence estimates, while opium consumption was linked to both lower awareness and control through lifestyle modifications prevalence estimates (Table 1).

**Table 1:** Baseline characteristics and questionnaire findings associated with hypertension awareness, treatment, and control through lifestyle modifications subgroups in individuals with hypertension.

Characteristics		All individuals with hypertension (*N* = 2333)			
		Overall	Awareness (*N* = 1958, 83.9%)	Treatment (*N* = 1896, 81.3%)	Control BP through lifestyle modifications (*N* = 79, 3.4%)
Demographics					
Age (year)		62.84 ± 7.58	62.96 ± 7.67	63.21 ± 7.71***	59.23 ± 6.23*
Female sex		1136 (48.69%)	1033 (52.76%)***	989 (52.16%)***	45 (56.96%)***
Body mass index (kg/m^2^)		29.37 ± 4.90	29.61 ± 4.87***	29.62 ± 4.86***	28.47 ± 5.09
Waist‐to‐hip ratio		0.98 ± 0.07	0.98 ± 0.07	0.98 ± 0.07	0.97 ± 0.07
Marital status	Singe	193 (8.27%)	181 (9.24%)***	174 (9.18%)**	8 (10.13%)**
	Married	2140 (91.73%)	1777 (90.76%)***	1722 (90.82%)**	71 (89.87%)**
Living status	Alone	127 (5.56%)	119 (6.21%)**	118 (6.36%)**	6 (7.59%)*
	With spouse/children	2157 (94.44%)	1797 (93.79%)**	1738 (93.64%)**	73 (92.41%)*
Education level	Illiterate	243 (10.42%)	214 (10.94%)***	212 (11.19%)**	10 (12.66%)
	Primary (elementary school)	1154 (49.49%)	1000 (51.10%)***	950 (50.13%)**	39 (49.37%)
	Middle	353 (15.14%)	275 (14.5%)***	268 (14.14%)**	11 (13.92%)
	Secondary (high school)	303 (12.99%)	249 (12.72%)***	244 (12.88%)**	10 (12.66%)
	Post‐secondary (≥ college)	279 (11.96%)	219 (11.19%)***	221 (11.66%)**	9 (11.39%)
Employment status	Employed	438 (18.78%)	353 (18.04%)*	330 (17.41%)***	13 (16.46%)***
	Retired/unemployed	1894 (81.22%)	1604 (81.96%)*	1565 (82.59%)***	66 (83.54%)***
Receiving unofficial care		601 (25.86%)	528 (27.04%)**	513 (27.16%)**	20 (25.32%)**
Comorbidities					
Diabetes mellitus		923 (39.77%)	831 (42.68%)***	826 (43.82%)***	33 (41.77%)**
Diabetes mellitus years		10.66 ± 8.19	10.79 ± 8.20	10.95 ± 8.22**	9.44 ± 6.22
Hypertension years		8.97 ± 7.47	8.96 ± 7.47	9.20 ± 7.50***	6.18 ± 6.05
Dyslipidemia		1445 (61.94%)	1275 (65.12%)***	1222 (64.45%)***	53 (67.09%)***
Dyslipidemia years		7.64 ± 6.58	7.87 ± 6.66***	7.89 ± 6.59***	8.96 ± 7.77*
Coronary heart disease		130 (5.60%)	113 (5.80%)	118 (6.26%)**	5 (6.33%)*
Chronic kidney disease		99 (4.27%)	95 (4.88%)**	97 (5.15%)***	4 (5.06%)
Smoking habits					
Smoking status	Current smoker	279 (11.96%)	218 (11.13%)**	210 (11.08%)**	8 (10.13%)***
	Ex‐smoker	308 (13.20%)	246 (12.56%)**	244 (12.87%)**	10 (12.66%)‡
	Never	1746 (74.84%)	1494 (76.30%)**	1442 (76.05%)**	61 (77.21%)***
Opium		125 (5.36%)	96 (4.91%)*	101 (5.33%)	3 (3.80%)**
Questionnaires–economic status					
Self‐expressed financial standing	High	13 (0.56%)	9 (0.46%)	11 (0.58%)	0
	Medium to high	154 (6.65%)	121 (6.22%)	121 (6.43%)	5 (6.33%)
	Medium	1148 (49.59%)	975 (50.10%)	948 (50.37%)	38 (48.10%)
	Low to medium	577 (24.92%)	487 (25.03%)	468 (24.87%)	20 (25.32%)
	Low	423 (18.27%)	354 (18.19%)	334 (17.75%)	16 (20.25%)
Physical and mental status					
Physical component score (PCS‐12)		45.16 ± 9.83	44.58 ± 9.95***	44.59 ± 9.95***	44.96 ± 10.17***
Poor physical condition (PCS‐12 ≤ 50)		1440 (61.72%)	1265(64.61%)***	1219 (64.29%)***	51 (64.56%)**
Mental component score (MCS‐12)		49.80 ± 9.76	49.45 ± 9.91***	49.46 ± 9.73***	48.46 ± 11.70***
Clinical depression (MCS‐12 ≤ 42)		459 (19.67%)	408 (20.84%)**	392 (20.68%)*	16 (20.25%)**
Life satisfaction					
Extremely satisfied (SWLS 31–35)		244 (10.46%)	193 (9.86%)	188 (9.92%)	8 (10.13%)*
Satisfied (SWLS 26–30)		999 (42.84%)	826 (42.19%)	809 (42.69%)	33 (41.77%)*
Slightly satisfied (SWLS 21–25)		562 (24.10%)	482 (24.62%)	461 (24.33%)	20 (25.32%)*
Neutral (SWLS 20)		82 (3.52%)	71 (3.63%)	67 (3.54%)	3 (3.80%)*
Slightly dissatisfied (SWLS 15–19)		266 (11.41%)	234 (11.95%)	228 (12.03%)	9 (11.39%)*
Dissatisfied (SWLS 10–14)		156 (6.69%)	131 (6.69%)	124 (6.54%)	5 (6.33%)*
Extremely dissatisfied (SWLS 5–9)		23 (0.99%)	21 (1.07%)	18 (0.95%)	1 (1.26%)*
Anxiety and depression					
Normal (HADS ≤ 7)		1754 (75.73%)	1439 (74.02%)***	1403 (74.51%)*	57 (72.16%)***
Borderline‐mild (HADS 8–10)		303 (13.08%)	266 (13.68%)***	258 (13.70%)*	11 (13.92%)***
Abnormal (HADS ≥ 11)		259 (11.18%)	239 (12.29%)***	222 (11.79%)*	11 (13.92%)***
Depression					
Normal (CES‐D < 10)		1948 (84.51%)	1608 (83.14%)***	1566 (83.61%)*	66 (83.54%)*
With depression symptoms (CES‐D ≥ 10)		357 (15.49%)	326 (16.86%)***	307 (16.39%)*	13 (16.46%)*

*Note:* Control BP refers to hypertensive patients with controlled blood pressure (systolic BP < 130 mmHg and diastolic BP < 80 mmHg), achieved either through lifestyle modifications or anti‐hypertensive medication.

Categorical data are reported as numbers (percentages in the column), and *p* values are calculated for subgroups in each column independently.

Abbreviations: BP, blood pressure; CES‐D, center for epidemiological studies‐depression scale; HADS, hospital anxiety and depression scale; SWLS, satisfaction with life scale.

* *p* value < 0.05

** *p* value < 0.01

*** *p* value < 0.001.

Individuals with clinical depression (MCS‐12 ≤ 42) exhibited higher awareness, treatment, and control through lifestyle modifications prevalence. However, based on HADS and CES‐D questionnaire results, borderline‐to‐mild anxiety (HADS: 8–10), abnormal anxiety (HADS ≥ 11), and the presence of depressive symptoms (CES‐D ≥ 10) were all associated with higher awareness, treatment, and control through lifestyle modifications prevalence estimates. Our analysis did not find any significant association between self‐reported financial standing or SWLS scores and any of these components (Table 1).

### 3.2 Control Among Treated

We evaluated controlled BP among individuals receiving anti‐hypertension medication and found that 1328 out of 1896 treated individuals (70.0%) had achieved target BP levels. BP control was more prevalent among younger individuals, women, those with a lower waist‐to‐hip ratio, retired or unemployed individuals, non‐smokers, and those without a history of opium use. Furthermore, no significant association was observed between BP control among treated individuals and any of the questionnaire responses (Table 2).

**Table 2:** Baseline characteristics and questionnaire findings associated with control among treated individuals receiving anti‐hypertension medication.

Characteristics		All treated individuals with hypertension (*N* = 1896)		
		Control (*N* = 1328, 70.0%)	Uncontrol (*N *= 568, 30.0%)	*p* value*
Demographics				
Age (year)		62.79 ± 7.46	64.19 ± 8.18	**< 0.001**
Female sex		750 (56.48%)	239 (42.08%)	**< 0.001**
Body mass index (kg/m^2^)		29.54 ± 4.82	29.80 ± 4.97	0.30
Waist‐to‐hip ratio		0.97 ± 0.07	0.99 ± 0.08	**< 0.001**
Marital status	Single	131 (9.86%)	43 (7.57%)	0.11
	Married	1197 (90.14%)	525 (92.43%)	
Living status	Alone	86 (6.64%)	32 (5.71%)	0.45
	With spouse/children	1210 (93.36%)	528 (94.29%)	
Education level	Illiterate	140 (10.55%)	72 (12.68%)	0.38
	Primary (elementary school)	679 (51.17%)	271 (47.71%)	
	Middle	183 (13.79%)	85 (14.96%)	
	Secondary (high school)	165 (12.43%)	79 (13.91%)	
	Post‐secondary (≥ college)	160 (12.06%)	61 (10.74%)	
Employment status	Employed	213 (16.05%)	117 (20.60%)	**0.017**
	Retired/unemployed	1114 (83.95%)	451 (79.40%)	
Receiving unofficial care		366 (27.62%)	147 (26.06%)	0.49
Comorbidities				
Diabetes mellitus		568 (43.03%)	258 (45.66%)	0.29
Diabetes mellitus years		10.86 ± 8.12	11.16 ± 8.46	0.63
Hypertension years		9.21 ± 7.64	9.18 ± 7.19	0.94
Dyslipidemia		867 (65.29%)	355 (62.50%)	0.25
Dyslipidemia years		7.89 ± 6.64	7.88 ± 6.47	0.99
Coronary heart disease		89 (6.74%)	29 (5.13%)	0.19
Chronic kidney disease		64 (4.58%)	33 (5.84%)	0.37
Smoking habits				
Smoking status	Current smoker	136 (10.24%)	74 (13.03%)	**0.001**
	Ex‐smoker	151 (11.37%)	93 (16.37%)	
	Never	1041 (78.39%)	401 (70.60%)	
Opium		56 (4.22%)	45 (7.92%)	**0.001**
Questionnaires–economic status				
Self‐expressed financial standing	High	7 (0.53%)	4 (0.71%)	0.64
	Medium to high	90 (6.82%)	31 (5.52%)	
	Medium	658 (49.85%)	290 (51.60%)	
	Low to medium	336 (25.45%)	132 (23.49%)	
	Low	229 (17.35%)	105 (18.68%)	
Physical and mental status				
Physical component score (PCS‐12)		44.60 ± 9.93	44.57 ± 10.02	0.94
Poor physical condition (PCS‐12 ≤ 50)		855 (64.38%)	364 (64.08%)	0.90
Mental component score (MCS‐12)		49.17 ± 9.84	50.14 ± 9.44	0.05
Clinical depression (MCS‐12 ≤ 42)		284 (21.39%)	108 (19.01%)	0.24
Life satisfaction				
Extremely satisfied (SWLS 31‐35)		120 (9.04%)	68 (11.97%)	0.28
Satisfied (SWLS 26‐30)		567 (42.73%)	242 (42.61%)	
Slightly satisfied (SWLS 21‐25)		337 (25.40%)	124 (21.83%)	
Neutral (SWLS 20)		42 (3.17%)	25 (4.40%)	
Slightly dissatisfied (SWLS 15‐19)		162 (12.21%)	66 (11.62%)	
Dissatisfied (SWLS 10‐14)		87 (6.56%)	37 (6.51%)	
Extremely dissatisfied (SWLS 5‐9)		12 (0.90%)	6 (1.06%)	
Anxiety and depression				
Normal (HADS ≤ 7)		965 (73.22%)	438 (77.52%)	0.07
Borderline‐mild (HADS 8–10)		184 (13.96%)	74 (13.10%)	
Abnormal (HADS ≥ 11)		169 (12.82%)	53 (9.38%)	
Depression				
Normal (CES‐D < 10)		1096 (83.60%)	470 (83.63%)	0.99
With depression symptoms (CES‐D ≥ 10)		215 (16.40%)	92 (16.37%)	

*Note:* Categorical data are reported as number (percentage in the column).

Abbreviations: CES‐D, center for epidemiological studies‐depression scale; HADS, hospital anxiety and depression scale; SWLS, satisfaction with life scale.

* *p* value < 0.05 was considered statistically significant and bolded.

### 3.3 Age–Sex Disparities

**Figure 2:**
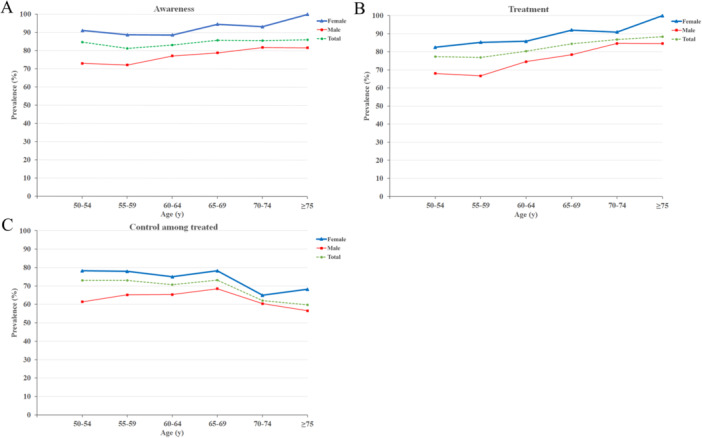
Prevalence of hypertension awareness stratified by age and sex (A); prevalence of hypertension treatment stratified by age and sex (B); and prevalence of control among treated individuals receiving anti‐hypertensive medications stratified by age and sex (C).

We analyzed hypertension awareness, treatment, and control among treated individuals across six different age categories and between women and men (Figure 2). Awareness prevalence remained relatively stable across age groups, with women consistently exhibiting higher awareness than men in all age groups—this difference was statistically significant across all age groups and exceeded 18% in the “50–54” and “≥ 75” age groups (Figure 2A; Supporting Information S1: Table 1). The prevalence of treatment increased with age in both women and men (Figure 2B). Women had a statistically significantly higher treatment prevalence than men across all age groups, except for the “50–54” age group, where the difference was not significant (Supporting Information S1: Table 1). BP control among treated individuals followed a consistent pattern up to the age of 70, but declined significantly thereafter. This decline was more pronounced in women, who experienced a −12.1% decrease in control prevalence and a −13.3% decrease in the control among treated prevalence (Figure 2C).

### 3.4 Multivariable Models

We applied multivariable logistic regression models to identify independent factors associated with hypertension awareness, treatment, control through lifestyle modifications, and control among treated individuals, adjusting for potential confounders within each subgroup (Table 3). Our analysis revealed that poor physical condition (PCS‐12 ≤ 50), as assessed using the SF‐12 questionnaire, was significantly associated only with higher awareness prevalence (OR: 1.34, 95% CI: 1.03–1.72, *p*: 0.03). Similarly, the presence of depressive symptoms (CES‐D ≥ 10), as determined by the CES‐D scale, was significantly associated with higher awareness prevalence (OR: 1.65, 95% CI: 1.08–2.52, *p*: 0.02). However, no significant associations were identified between the results from other questionnaires and hypertension awareness, treatment, control through lifestyle modifications, or control among treated individuals in the models (all *p *> 0.05; Table 3). Details on baseline characteristics and questionnaire findings—stratified by physical condition (based on PCS‐12) and depressive symptoms (based on CES‐D)—among individuals with hypertension are presented in Supporting Information S1: Tables 2 and 3.

**Table 3:** Multivariable logistic regression models for predicting hypertension awareness, treatment, and control through lifestyle modifications, and control among treated individuals in patients with hypertension.

Outcome	Predictive factors (questionnaire)	Multivariable model**		
		OR	95% CI	*p* value*
Awareness	Poor physical condition (PCS‐12 ≤ 50)	1.34	1.03–1.72	**0.03**
	Clinical depression (MCS‐12 ≤ 42)	1.35	0.96–1.91	0.08
	Anxiety and depression (HADS > 7)	1.25	0.85–1.86	0.25
	Depressive symptoms (CES‐D ≥ 10)	1.65	1.08–2.52	**0.02**
Treatment	Poor physical condition (PCS‐12 ≤ 50)	1.06	0.84–1.35	0.61
	Clinical depression (MCS‐12 ≤ 42)	1.28	0.94–1.74	0.11
	Anxiety and depression (HADS > 7)	1.28	0.89–1.84	0.18
	Depressive symptoms (CES‐D ≥ 10)	1.09	0.77–1.53	0.63
Control through lifestyle modifications	Poor physical condition (PCS‐12 ≤ 50)	1.05	0.81–1.29	0.68
	Clinical depression (MCS‐12 ≤ 42)	1.13	0.92–1.34	0.20
	Anxiety and depression (HADS > 7)	1.08	0.77–1.39	0.61
	Depressive symptoms (CES‐D ≥ 10)	1.02	0.68–1.36	0.91
Control among treated	Poor physical condition (PCS‐12 ≤ 50)	0.92	0.75–1.15	0.47
	Clinical depression (MCS‐12 ≤ 42)	0.99	0.77–1.28	0.96
	Anxiety and depression (HADS > 7)	0.97	0.72–1.32	0.87
	Depressive symptoms (CES‐D ≥ 10)	0.84	0.64–1.11	0.21

Abbreviations: BMI, body mass index; CES‐D, center for epidemiological studies‐depression scale; DM, diabetes mellitus; HADS, hospital anxiety and depression scale; MCS‐12, mental component score from SF‐12; OR, odds ratio; PCS‐12, physical component score from SF‐12.

* *p* value < 0.05 was considered statistically significant and bolded.

** Significant variables in univariate analysis were considered as potential confounders and adjusted in each model (Awareness models were adjusted with age, sex, BMI, education level, DM, dyslipidemia; treatment models were adjusted with age, sex, BMI, DM; control models were adjusted with age, sex, employment status; control among treated models were adjusted with age, sex, employment status).

## 4 Discussion

In the present study, we investigated hypertension management findings from the ACSA recruitment phase. Among hypertensive individuals, 84% were aware, 81% were treated with anti‐hypertensive medication, and 60% had controlled BP through lifestyle modifications or anti‐hypertensive medications. Multivariable regression analysis revealed that, after adjusting for potential confounders, poor physical condition (evaluated using the SF‐12 questionnaire) and the presence of depressive symptoms (assessed using the CES‐D scale) were independently associated with higher hypertension awareness prevalence. Furthermore, our findings demonstrated that women consistently exhibited a higher prevalence of hypertension awareness, treatment, and control compared with men.

Hypertension is a significant concern among older adults, characterized by complex pathophysiological changes in the cardiovascular system, such as arterial stiffening and alterations in the renin‐angiotensin‐aldosterone system [23]. Aging is commonly associated with a gradual increase in BP, particularly SBP, which typically rises until approximately 70–80 years of age. Conversely, DBP tends to increase until around 50–60 years of age before it starts to decline, leading to a progressive increase in pulse pressure beyond 60 years of age [24]. As a result, isolated systolic hypertension is emerging as the predominant type of elevated blood pressure observed in older individuals. However, in this study, systolic‐diastolic hypertension was the most prevalent form of hypertension, exhibiting more than twice the prevalence of isolated systolic hypertension (47% vs. 21%). We hypothesize that this higher prevalence may be linked to the revised hypertension criteria in the 2017 ACC/AHA guidelines, which introduced lower cut‐off points, particularly for DBP [25, 26]. This age‐related increase in blood pressure has not been observed in certain populations with traditional lifestyles, suggesting that lifestyle and environmental factors may play a more significant role than aging itself [23]. Furthermore, coexisting comorbidities like diabetes, dyslipidemia, and chronic kidney disease can notably influence age‐related BP variations [27].

Recent studies indicate significant global disparities in the prevalence and control of hypertension, with this gap appearing to widen over time [28]. Compared to high‐income countries (HICs), LMICs consistently report a higher prevalence of hypertension but exhibit lower levels of awareness, treatment, and control. This disparity is particularly evident among older adults, with HICs reporting 34.3% higher awareness, 43.7% higher treatment, and 17.3% higher control prevalences compared with LMICs [29]. A key factor contributing to this disparity could be the limited health infrastructure and resources in LMICs, which make their primary healthcare systems less equipped to address the hypertension epidemic effectively. According to health and retirement studies in 2012, adults aged ≥ 50 in the United States reported the highest prevalence of hypertension awareness (85.1%), treatment (78.5%), and control (52.2%) [29]. In this study, 84% of individuals with hypertension were aware of their condition, and 81% were receiving anti‐hypertensive medication. These findings indicate notable improvements in awareness and treatment prevalence compared to prior studies conducted in Iran [6, 7, 9, 30]. For instance, a 2022 population‐based cohort study in Tehran (the capital of Iran) reported prevalence estimates of 76.0% for awareness, 62.5% for treatment, and 45.4% for control among individuals aged ≥ 55 [6]. Similarly, a 2018 study from Isfahan documented prevalence estimates of 77.6% for awareness, 94.1% for treatment, and 58.0% for control among individuals aged ≥ 50 [9]. The high levels of awareness and treatment in our study may be partially explained by an increased frequency of medical visits and more intensive medical care during the COVID‐19 pandemic.

Our study revealed that specific demographic characteristics were significantly associated with higher awareness and treatment of hypertension. These factors included women, being single, living alone, being retired or unemployed, receiving informal care, and having a history of diabetes mellitus or dyslipidemia. At younger ages, women typically exhibit lower blood pressure and a lower risk of developing hypertension compared with men. However, with aging, blood pressure gradually increases in both sexes, with a more pronounced rise observed in postmenopausal women. This pattern may explain the higher prevalence of hypertension among older women [29]. Moreover, it's worth noting that men are generally less likely than women to seek medical advice or utilize healthcare services, which could contribute to lower awareness and treatment prevalence among men [31]. Additionally, individuals leading busy lifestyles exhibited lower awareness, treatment, and control prevalences. These individuals may prioritize work and other commitments over routine health check‐ups and preventive care. Targeted public health interventions should address this group to enhance hypertension management and improve overall cardiovascular health.

Our study identified a significant association between depressive symptoms and reduced hypertension awareness among older adults, as assessed by the CES‐D scale (OR: 1.65, 95% CI: 1.08–2.52). These findings align with previous research. The HOPE Asia Network reported that older adults with hypertension frequently experience depression, which can overshadow their awareness and management of hypertension [32]. Institutional and social factors, such as limited social support, may exacerbate depressive symptoms, further reducing hypertension recognition [33]. Additionally, cultural differences may influence the relationship between depression and hypertension awareness, affecting health‐seeking behaviors and self‐care practices. For healthcare providers, identifying and addressing depressive symptoms in older hypertensive patients is crucial, as depression presents a significant barrier to medication adherence and effective blood pressure management [23]. Integrating mental health screening into routine hypertension care could improve both awareness and treatment outcomes in this population.

### 4.1 Limitations

There are certain limitations to this study that warrant careful consideration. First, as a cross‐sectional study, it prevents the establishment of any causal relationships between the variables. To obtain more robust insights, longitudinal studies are recommended. Second, the sample was restricted to participants from Ardakan City, which may limit the generalizability of the findings to other populations or ethnicities. Third, the reliance on self‐reported data introduces the potential for recall bias, which could impact the accuracy of the results. Furthermore, health conditions were identified based on past medical records and medication use, which might overlook undiagnosed cases. Fourth, while individuals diagnosed with dementia or cognitive impairments were excluded, undetected mild cognitive deficits could still influence the accuracy of responses [34]. Fifth, there are still some unmeasured confounders, such as dietary habits or family health history, that may have contributed to the observed associations but were not evaluated in this study. Despite these limitations, the current research offers valuable insights into the health determinants affecting older adults in Ardakan City and underscores the need for further research to strengthen and expand upon these findings.

## 5 Conclusion

This study reveals significant insights into hypertension management among the elderly in Ardakan City, Iran, highlighting that while 84% of hypertensive individuals were aware of their condition and 81% were treated with anti‐hypertensive medications, only 60% achieved controlled blood pressure through lifestyle modifications or anti‐hypertensive medications. Women consistently demonstrated higher prevalence estimates for awareness, treatment, and control compared with men. Factors such as poor physical condition and depressive symptoms were strongly associated with higher awareness prevalence estimates. These findings underscore the need for targeted interventions that address both physical and mental health aspects to improve hypertension management in this vulnerable population.

## Author Contributions

Sina Kazemian and Elham Hooshmand designed the study. Elham Hooshmand contributed to data collection and provided critical feedback on the manuscript. Sina Kazemian, Fatemeh‐Sadat Tabatabaei, Amirali Azimi, Isa Akbarzade, and Elham Hooshmand assisted with data preparation and data analysis, and contributed to writing and critically reviewing multiple drafts of the manuscript. All authors read and approved the final version of the manuscript. Ms. Elham Hooshmand had full access to all the data in the study and takes responsibility for the integrity of the data and the accuracy of the data analysis.

## Conflicts of Interest

The authors declare no conflicts of interest.

## Transparency Statement

The corresponding author, Elham Hooshmand, affirms that this manuscript is an honest, accurate, and transparent account of the study being reported; that no important aspects of the study have been omitted; and that any discrepancies from the study as planned (and, if relevant, registered) have been explained.

## Supporting information


**Supplementary Table 1:** Prevalence of hypertension awareness, treatment, control through lifestyle modifications, and control among treated within age‐sex subgroups among individuals with hypertension. **Supplemental Table 2:** Baseline characteristics and questionnaire findings associated with physical condition among individuals with hypertension. **Supplemental Table 3:** Baseline characteristics and questionnaire findings associated with depressive symptoms among individuals with hypertension.

## Data Availability

The data sets analyzed during the current study are available from the corresponding author on reasonable request.
