# Supplementary material for: Awareness, Treatment, and Control of Hypertension Among Hypertensive Older Adults in Iran: A Cross‐Sectional Study From the Ardakan Cohort Study on Aging
Source: Health Sci Rep. 2025 Dec 15;8(12):e71660. doi: 10.1002/hsr2.71660 (PMC12705481; doi:10.1002/hsr2.71660)
Supplement: Supplementary file 1 — Supplementary Table 1: Prevalence of hypertension awareness, treatment, control through lifestyle modifications, and control among treated within age‐sex subgroups among individuals with hypertension. Supplemental Table 2: Baseline characteristics and questionnaire findings associated with physical condition among individuals with hypertension. Supplemental Table 3: Baseline characteristics and questionnaire findings associated with depressive symptoms among individuals with hypertension. [file HSR2-8-e71660-s001.pdf]

Awareness, Treatment, and Control of Hypertension among Hypertensive Older Adults in Iran: A Cross-Sectional Study from the Ardakan Cohort Study on Aging

Supplemental Material

Supplementary Table 1. Prevalence of hypertension awareness, treatment, control, and control among treated within age-sex subgroups among individuals with hypertension. 2

Supplemental Table 2. Baseline characteristics and questionnaire findings associated with physical condition among individuals with hypertension 3

Supplemental Table 3. Baseline characteristics and questionnaire findings associated with depressive symptoms among individuals with hypertension..6

Supplementary Table 1. Prevalence of hypertension awareness, treatment, control through lifestyle modifications, and control among treated within age-sex subgroups among individuals with hypertension

	**Awareness**				**Treatment**				**Control through lifestyle modifications**				**Control among treated**			
**Age (year)**	Total	Women	Men	*P**	Total	Women	Men	*P**	Total	Women	Men	*P**	Total	Women	Men	*P**
**50-54**	292 (84.6%)	203 (91.0%)	89 (72.9%)	**<0.001**	267 (77.4%)	184 (82.5%)	83 (68.0%)	**0.002**	22 (6.4%)	18 (8.1%)	4 (3.3%)	0.075	195 (73.0%)	144 (78.3%)	51 (61.4%)	**0.004**
**55-59**	430 (81.3%)	259 (88.7%)	171 (72.1%)	**<0.001**	407 (76.9%)	249 (85.3%)	158 (66.7%)	**<0.001**	23 (4.3%)	15 (5.1%)	8 (3.4%)	0.34	297 (73.0%)	194 (77.9%)	103 (65.2%)	**0.005**
**60-64**	465 (83.0%)	256 (88.6%)	209 (77.1%)	**<0.001**	450 (80.4%)	248 (85.8%)	202 (74.5%)	**0.001**	21 (3.7%)	10 (3.5%)	11 (4.1%)	0.71	318 (70.7%)	186 (75.0%)	132 (65.3%)	**0.02**
**65-69**	390 (85.7%)	189 (94.5%)	201 (78.8%)	**<0.001**	384 (84.4%)	184 (92.0%)	200 (78.4%)	**<0.001**	7 (1.5%)	3 (1.5%)	4 (1.6%)	0.93	281 (73.2%)	144 (78.3%)	137 (68.5%)	**0.03**
**70-74**	226 (85.6%)	82 (93.2%)	144 (81.8%)	**0.01**	229 (86.7%)	80 (90.9%)	149 (84.7%)	0.16	4 (1.5%)	2 (2.3%)	2 (1.1%)	0.44	142 (62.0%)	52 (65.0%)	90 (60.4%)	0.49
**≥75**	155 (86.1%)	44 (100%)	111 (81.6%)	**0.002**	159 (88.3%)	44 (100%)	115 (84.6%)	**0.006**	2 (1.1%)	0	2 (1.5%)	0.41	95 (59.7%)	30 (68.2%)	65 (56.5%)	0.18

* P-value <0.05 was considered statistically significant and bolded.

Supplemental Table 2. Baseline characteristics and questionnaire findings associated with physical condition among individuals with hypertension

Characteristics		All individuals with hypertension (N=2333)		
		**Poor physical condition (PCS-12 ≤50) (N=1440, 61.7%)**	**Good physical condition (PCS-12 >50) (N=893, 38.3%)**	**P-value***
**Demographics**				
Age (year)		63.34±0.20	62.02±0.24	**<0.001**
Women, %		832 (57.8%)	304 (34.0%)	**<0.001**
Body mass index (kg/m2)		30.10±0.14	28.28±0.16	**<0.001**
Marital status	Single	146 (10.14%)	47 (5.26%)	**<0.001**
	Married	1294 (89.86%)	846 (94.74%)	
Living status	Alone	97 (6.87%)	30 (3.44%)	**0.001**
	With spouse/children	1315 (93.13%)	842 (96.56%)	
Education level	Illiterate	177 (12.30%)	66 (7.39%)	**<0.001**
	Primary (elementary school)	804 (55.87%)	350 (39.19%)	
	Middle	206 (14.32%)	147 (16.46%)	
	Secondary (high school)	146 (10.15%)	157 (17.58%)	
	Post-secondary (≥college)	106 (7.37%)	173 (19.37%)	
Employment status	Employed	241 (16.75%)	197 (22.06%)	**0.001**
	Retired/unemployed	1198 (83.25%)	696 (77.94%)	
Receiving unofficial care		494 (34.40%)	107 (12.05%)	**<0.001**
**Comorbidities**				
Diabetes mellitus		660 (46.09%)	263 (29.58%)	**<0.001**
Diabetes mellitus years		10.88±0.32	10.14±0.49	0.22
Hypertension years		9.51±0.22	7.97±0.26	**<0.001**
Dyslipidemia		974 (67.64%)	471 (52.74%)	**<0.001**

Dyslipidemia years		8.09±0.22	6.72±0.28	**0.001**
Coronary heart disease		83 (5.79%)	47 (5.29%)	0.61
Chronic kidney disease		74 (5.17%)	25 (2.81%)	**0.006**
**Smoking habits**				
Smoking status	Current smoker	153 (10.62%)	126 (14.11%)	**0.001**
	Ex-smoker	172 (11.94%)	136 (15.23%)	
	Never	1115 (77.43%)	631 (70.66%)	
Opium		69 (4.79%)	56 (6.29%)	0.12
**Questionnaires-Economic status**				
Self-expressed financial standing	High	5 (0.35%)	8 (0.90%)	**<0.001**
	Medium to high	60 (4.19%)	94 (10.63%)	
	Medium	711 (49.69%)	437 (49.43%)	
	Low to medium	362 (25.30%)	215 (24.32%)	
	Low	293 (20.48%)	130 (14.71%)	
**Mental status**				
Mental component score (MCS-12)		50.05±0.26	49.40±0.32	0.12
**Life satisfaction**				
Extremely satisfied (SWLS 31-35)		107 (7.43%)	137 (15.36%)	**<0.001**
Satisfied (SWLS 26-30)		595 (41.32%)	404 (45.29%)	
Slightly satisfied (SWLS 21-25)		357 (24.79%)	205 (22.98%)	
Neutral (SWLS 20)		62 (4.31%)	20 (2.24%)	
Slightly dissatisfied (SWLS 15-19)		192 (13.33%)	74 (8.30%)	
Dissatisfied (SWLS 10-14)		110 (7.64%)	46 (5.16%)	
Extremely dissatisfied (SWLS 5-9)		17 (1.18%)	6 (0.67%)	
**Anxiety and depression**				
Normal (HADS ≤7)		1006 (70.20%)	748 (84.71%)	**<0.001**
Borderline-mild (HADS 8-10)		221 (15.42%)	82 (9.29%)	
Abnormal (HADS ≥11)		206 (14.38%)	53 (6.00%)	
**Depression**				

Normal (CES-D <10)	1144 (80.11%)	804 (91.68%)	**<0.001**
With depression symptoms (CES-D ≥10)	284 (19.89%)	73 (8.32%)	

Categorical data are reported as number (percentage in the column).
* P-value <0.05 was considered statistically significant and bolded.
Abbreviations: CES-D: center for epidemiologic studies-depression scale, HADS: hospital anxiety and depression scale, SWLS: satisfaction with life scale.

Supplemental Table 3. Baseline characteristics and questionnaire findings associated with depressive symptoms among individuals with hypertension

**Characteristics**		**All individuals with hypertension (N=2333)**		
		**Presence of depressive symptoms (CES-D ≥10) (N=459, 19.7%)**	**Absence of depressive symptoms (CES-D <10) (N=1874, 80.3%)**	**P-value***
**Demographics**				
Age (year)		61.67±0.33	63.12±0.18	**<0.001**
Women, %		302 (65.80%)	834 (44.50%)	**<0.001**
Body mass index (kg/m2)		29.69±0.25	29.29±0.12	0.14
Marital status	Single	59 (12.85%)	134 (7.15%)	**<0.001**
	Married	400 (87.15%)	1740 (92.85%)	
Living status	Alone	39 (8.74%)	88 (4.79%)	**0.001**
	With spouse/children	407 (91.26%)	1750 (95.21%)	
Education level	Illiterate	60 (13.07%)	183 (9.77%)	**<0.001**
	Primary (elementary school)	259 (56.43%)	895 (47.78%)	
	Middle	57 (12.42%)	296 (15.80%)	
	Secondary (high school)	52 (11.33%)	251 (13.40%)	
	Post-secondary (≥college)	31 (6.75%)	248 (13.24%)	
Employment status	Employed	72 (15.69%)	366 (19.54%)	0.06
	Retired/unemployed	387 (84.31%)	1507 (80.46%)	
Receiving unofficial care		165 (36.26%)	436 (23.33%)	**<0.001**
**Comorbidities**				
Diabetes mellitus		195 (42.86%)	728 (39.01%)	0.13
Diabetes mellitus years		11.15±0.65	10.54±0.29	0.35
Hypertension years		9.05±0.34	8.94±0.19	0.79
Dyslipidemia		326 (71.02%)	1119 (59.71%)	**<0.001**

Dyslipidemia years		8.00±0.35	7.54±0.20	0.26
Coronary heart disease		26 (5.70%)	104 (5.57%)	0.91
Chronic kidney disease		21 (4.62%)	78 (4.18%)	0.68
**Smoking habits**				
Smoking status	Current smoker	54 (11.76%)	225 (12.01%)	**0.022**
	Ex-smoker	43 (9.37%)	265 (14.14%)	
	Never	362 (78.87%)	1384 (73.85%)	
Opium		21 (4.59%)	104 (5.55%)	0.41
**Questionnaires-Economic status**				
Self-expressed financial standing	High	1 (0.22%)	12 (0.64%)	**<0.001**
	Medium to high	23 (5.08%)	131 (7.04%)	
	Medium	186 (41.06%)	962 (51.66%)	
	Low to medium	126 (27.81%)	451 (24.22%)	
	Low	117 (25.83%)	306 (16.43%)	
**Physical and mental status**				
Physical component score (PCS-12)		44.79±0.50	45.25±0.22	0.37
Mental component score (MCS-12)		34.25±0.31	53.62±0.13	**<0.001**
**Life satisfaction**				
Extremely satisfied (SWLS 31-35)		19 (4.14%)	225 (12.01%)	**<0.001**
Satisfied (SWLS 26-30)		109 (23.75%)	890 (47.52%)	
Slightly satisfied (SWLS 21-25)		109 (23.75%)	453 (24.19%)	
Neutral (SWLS 20)		27 (5.88%)	55 (2.94%)	
Slightly dissatisfied (SWLS 15-19)		99 (21.57%)	167 (8.92%)	
Dissatisfied (SWLS 10-14)		81 (17.65%)	75 (4.00%)	
Extremely dissatisfied (SWLS 5-9)		15 (3.27%)	8 (0.43%)	
**Anxiety and depression**				
Normal (HADS ≤7)		203 (44.81%)	1551 (83.25%)	**<0.001**
Borderline-mild (HADS 8-10)		117 (25.83%)	186 (9.98%)	
Abnormal (HADS ≥11)		133 (29.36%)	126 (6.76%)	

Categorical data are reported as number (percentage in the column).

* P-value <0.05 was considered statistically significant and bolded.
Abbreviations: CES-D: center for epidemiologic studies-depression scale, HADS: hospital anxiety and depression scale, SWLS: satisfaction with life scale.
